# How do social norms influence the sexual and reproductive health-related attitudes and behaviours of very young adolescents in Sub-Saharan Africa? A scoping review

**DOI:** 10.1186/s12889-025-25736-z

**Published:** 2025-11-22

**Authors:** Fardawsa A. Ahmed, Owen Nyamwanza, Alice Ladur, Jermaine M. Dambi, Frances M. Cowan, Webster Mavhu

**Affiliations:** 1 Department of International Public Health, Liverpool School of Tropical Medicine, Liverpool, UK; 2 Centre for Sexual Health and HIV/AIDS Research (CeSHHAR), Harare, Zimbabwe; 3 Department of Rehabilitation Sciences, Faculty of Medicine and Health Sciences, University of Zimbabwe, Harare, Zimbabwe; 4 Friendship Bench, Harare, Zimbabwe

**Keywords:** Social norms, Very young adolescents, Sexual and reproductive health, Gender-responsive, Sub-Saharan Africa

## Abstract

**Background:**

In Sub-Saharan Africa (SSA), very young adolescents (aged 10–14 years) have the worst sexual and reproductive health (SRH) outcomes of this age group worldwide due to a range of factors, including social and gender norms. However, in this setting, SRH programming often focuses on older adolescents (aged 15–19 years), overlooking very young adolescents. This scoping review sought to explore how social and gender norms influence very young adolescents’ SRH-related attitudes and behaviours in SSA and draw inferences for culturally sensitive, gender-responsive interventions.

**Methods:**

The review followed the five-step framework developed by Arksey and O’Malley: (1) defining the research question, (2) identifying relevant studies, (3) selecting studies, (4) charting the data, and (5) collating, summarising, and reporting the results. We searched four databases (MEDLINE, CINAHL, Global Health, and Web of Science) for peer-reviewed articles published between January 1, 2000 and December 31, 2024.

**Results:**

We identified 24 studies: *n* = 11 (46%) were entirely qualitative, *n* = 8 (33%) exclusively quantitative, and three other quantitative studies incorporated qualitative components. Two studies used participatory techniques. Studies were from nine countries in SSA. Identified norms included those relating to menstruation, puberty, circumcision, romantic relationships and gender stereotypes. Social norms led to very young adolescents’ limited SRH knowledge and access, and behaviours and practices that heightened very young adolescents’ vulnerabilities and poor SRH outcomes. Evaluations of interventions to shift these norms reported mixed results, and highlighted the importance of adapting gender-responsive/gender-transformative interventions to the local context.

**Conclusions:**

Scoping review findings affirm the importance of intervening in very young adolescence to positively influence social and gender norms. The review underscores the importance of tailored, multifaceted, culturally sensitive, gender-responsive/gender-transformative interventions to improve young adolescents’ SRH-related attitudes and behaviours in Sub-Saharan Africa.

**Supplementary Information:**

The online version contains supplementary material available at 10.1186/s12889-025-25736-z.

## Background

The World Health Organisation (WHO) defines “adolescents” as individuals in the 10–19 years age group [1]. Worldwide, there are ∼1.3 billion adolescents, accounting for 16% of the global population [2]. About half are classified as very young adolescents (aged 10–14 years), with around 90% of very young adolescents residing in low- and middle-income countries [2]. In Africa, the number of young people is projected to double in the next 30 years, rendering (very young) adolescents a particularly important group [3]. As predicted by the World Bank, Africa’s ability to benefit from the projected population growth directly depends on the health and well-being of today’s adolescents and the educational opportunities available to them [3]. It is therefore crucial to invest in the health of very young adolescents across a wide range of outcomes, which can lead to improved productivity and economic gains [4].

In Sub-Saharan Africa (SSA), it is particularly critical to focus on very young adolescents’ sexual and reproductive health (SRH) given adolescents in this region have the worst SRH outcomes of this age group worldwide [5, 6]. Here, rates of adolescent childbearing among girls (15–19 years-old) are among the highest globally, ranging from 18% to 40% [7]. While adolescent birth rates have declined worldwide, in 2021, SSA still recorded more than double the global average, with over 100 births per 1,000 adolescent girls [7]. Unmarried girls also have the highest rates of abortion and abortion-related morbidity and mortality of any region [5, 6]. Additionally, key SRH service indicators such as HIV testing, contraceptive use and antenatal visits are poorer than in any other WHO region [8, 9]. Further, even before the current international HIV funding crisis [10–12], adolescent girls in SSA accounted for 75% of new HIV infections globally [13].

These SRH challenges are due to a range of intersecting factors, including social and gender norms [14, 15]. Social norms are unwritten, informal rules that determine how groups of people ought to behave in certain situations [4, 16, 17]. Gender norms are a subtype of social norms that dictate how males and females ought to behave [4, 16–19]. Social and gender norms are accompanied by sets of “sanctions” for both norm adherents/abiders and violators [17, 20]. Particularly in SSA, social and gender norms reflect the predominantly patriarchal character of most societies [17, 19, 21]. Patriarchal structures generally confer power on males to control resources and dominate females, leading to inequitable social and gender norms [22]. There are several other intertwined norms related to control of female sexuality, virginity, fertility, childbearing, and family planning use [21, 23, 24]. These mostly impede girls’ and women’s healthy SRH behaviours and service-seeking, and contribute to poorer SRH outcomes.

While gender socialisation begins in childhood, it intensifies in very young adolescence (10–14 years) and solidifies in later adolescence (≥ 15 years) [25–27]. However, the plasticity of the very young adolescent brain offers an important opportunity to shape behaviour and manipulate social constructs [26]. Intervening in very young adolescence, when perceptions and attitudes are still malleable, provides the opportunity to challenge inequitable gender stereotypes before they are solidified and become less difficult to change [4]. Once positive social norms are inculcated, their impact has important consequences for the well-being and SRH of very young adolescents both now and over their life course [4, 16]. Of note, gender-transformative approaches, that work with groups of males and females to promote critical reflection on harmful gender norms and unequal power dynamics, and build relationship skills such as communication and conflict resolution [19], can empower very young adolescents to challenge harmful norms.

In SSA though, SRH programmes have mostly focused on older adolescents (15–19 years) [28], due to at least two reasons. Firstly, very young adolescents are less prone to risk-taking than older adolescents [29]. Secondly, there exist widespread misperceptions that very young adolescents are too young to be sexually active or to need SRH information [30–32], which serve as obstacles to both programme and research development. Consequently, a few initiatives have focused on very young adolescents, with the Global Early Adolescent Study (GEAS) [33] being an exception.

The GEAS is a multi-country, longitudinal initiative exploring very young adolescents’ perceptions of the gender norms that regulate their behaviour, how they form their own beliefs about gender, and how these beliefs align with community social norms, including in four SSA countries: Democratic Republic of Congo, Kenya, Malawi and South Africa [34]. An important GEAS observation has been that intervention effects differ by context, and results can be highly contextual, even for seemingly similar settings [35]. Indeed, there is increasing recognition that African masculinities are produced in varying and unique contexts of intersections (including ethnicity and sexuality) [36]. Therefore, whilst some core intervention components can be implemented in different settings, there is a need for context-specific adaptations.

To inform such adaptations, we first need to understand the intersecting and contextual factors across various settings. We therefore conducted a scoping review to explore a body of literature to identify what is known about how social and gender norms influence very young adolescents’ SRH-related attitudes and behaviours in SSA to inform culturally sensitive, gender-responsive interventions in Zimbabwe and the region more widely.

## Methods

### Study design

We conducted an initial search in publicly available registries, including PROSPERO and Open Science Framework (OSF) for planned or ongoing reviews, and did not identify any focusing on this topic. We developed a protocol to guide our review, uploaded it on OSF (www.10.17605/OSF.IO/EW7S5) and subsequently published it [4]. We conducted the review according to the Preferred Reporting Items for Systematic Reviews and Meta-Analyses Extension for Scoping Reviews (PRISMA-ScR) guidelines (S1 File) [37]. We employed the scoping review framework developed by Arksey and O’Malley, whose stages include: (1) defining the research question, (2) identifying relevant studies, (3) selecting studies, (4) charting the data, and (5) collating, summarising and reporting the results [38].

### Stage 1: defining the research question

We used the Population–Concept–Context (PCC) framework [39] (Table 1) to inform the main review question.

**Table 1:** PCC framework

Framework item	Item components
Population(s)	-very young adolescents, both male and female (aged 10–14 years)
Concept(s)	-social norms/attitudes or perceptions of social norms -gender norms/attitudes or perceptions of gender norms -sexual and reproductive health
Context(s)	-Sub-Saharan Africa, the area and regions of the continent of Africa that lie south of the Sahara, including Central, East, South and West Africa

*The main review question was “How do social and gender norms influence the SRH-related attitudes and behaviours of very young adolescents (aged 10–14) in SSA?”* A sub-question was *“To what extent is the influence similar or different for girls and boys?”* These questions enabled us to map the range of relevant literature around these aspects and inform the direction of future research and programming.

### Stage 2: identifying relevant studies

We developed a search strategy to identify relevant studies from 1 January 2000 to 31 December 2024 (inclusive). We did not apply any language restrictions. We chose 2000 as our baseline year as this is when research on social and gender norms, very young adolescents and SRH issues in SSA intensified, including as part of the Millennium/Sustainable Development Goals’ global health programmes [40]. For example, Sustainable Development Goal (SDG) 5 recognises gender equality as a fundamental human right and a necessary foundation for a prosperous, peaceful and sustainable world [41].

We identified studies relevant to this review by searching electronic databases of published literature in PubMed/MEDLINE, CINAHL, Global Health and Web of Science. We carefully selected these databases for their comprehensiveness in covering the area under research. Our familiarity with these databases also enhanced efficiency in the search process. With the help of a librarian, we first developed the MEDLINE search strategy (S2 File) and later adapted it for the other three databases. Overall, the general search strategy (previously published [4]) was informed by the PCC framework, as shown in Table 2.

**Table 2:** Search strategy

Concept	Search Terms
Social norms	“Social norms” OR “Cultural pluralism” OR “Social norms attitudes” OR “Social norms perceptions” OR “sociocultural restrictions” OR “protective behaviours” OR “African culture” OR;
Gender norms	“Gender” OR “Gender norms” OR “Gender norms attitudes” OR “Gender norms perceptions” OR “Gender roles” OR “Gender attitudes” OR “Gender practices” OR “Gender Transform*” AND;
Sexual and reproductive health	“Sexual behaviour” OR “sexual health” OR “Youth sexual behaviour” OR “Attitudes toward sex” OR “Sexuality” OR “Sexual Health -- In Adolescence” OR “Reproductive Health -- In Adolescence” OR “contraceptives” OR “family planning” OR “Protected sex” OR “HIV” OR “STI” OR “sexual transmitted diseases” OR “pregnancy” AND;
Very young adolescents	“Youth” OR “young person” OR “minor” OR “10–14 years old” OR “adolescent” OR “teenage” OR “young adolescent*” OR “Very young adolescents” OR “early adolescent” AND;
Sub-Saharan Africa	“Angola” OR “Benin” OR “Botswana” OR “Burkina Faso” OR “Burundi” OR “Cameroon” OR “Cape Verde” OR “Central African Republic” OR “CHAD” OR “Comoros” OR “Congo” OR “Congo Democratic Republic” OR “Djibouti” OR “Equatorial Guinea” OR “Eritrea” OR “Ethiopia” OR “Gabon” OR “Gambia” OR “Ghana” OR “Guinea” OR “Guinea-Bissau” OR “Cote d’Ivoire” OR “Ivory Coast” OR “Kenya” OR “Lesotho” OR “Liberia” OR “Madagascar” OR “Malawi” OR “Mali” OR “Mozambique” OR “Namibia” OR “Niger” OR “Nigeria” OR “Sao Tome and Principe” OR “Rwanda” OR “Senegal” OR “Seychelles” OR “Sierra Leone” OR “Somalia” OR “South Africa” OR “South Sudan” OR “Sudan” OR “Swaziland” OR “Tanzania” OR “Togo” OR “Uganda” OR “Zambia” OR “Zimbabwe” OR “Africa, South of the Sahara” OR “Sub-Saharan Africa’’

On 19th April 2024, the lead author (FAA) tested a general search strategy by running it using a Boolean search on the Discover platform, combining the search terms from the PCC framework using “AND” and separating different terms using “OR”. She then searched each of the four databases (PubMed/MEDLINE, CINAHL, Global Health and Web of Science) using PCC framework concepts and their synonyms to identify specific terms used in these databases. She then combined the specific terms with free text terms contributed by co-authors. Reviewers then downloaded and imported search results into EndNote 20. After removing duplicates in EndNote 20, they exported the articles to Covidence, a collaborative software. The search was closed on 31 st December 2024.

### Stage 3: study selection

Reviewers removed additional duplicates in Covidence and proceeded with study selection, which consisted of two levels of screening, that is, title and abstract review and full-text review. FAA and a co-reviewer (ON) conducted the two levels of screening using Covidence software. The two reviewers reviewed any discordant full-text articles a second time and further disagreements about study eligibility at the full-text review stage were resolved through discussion with a senior researcher (WM) until full consensus was reached.

### Inclusion and exclusion criteria

Guided by the PCC framework, we included studies meeting all the criteria in each category outlined in Table 3. We included studies that employed quantitative, qualitative or mixed methods. We excluded reviews (scoping, narrative, systematic, meta-analyses, etc.), personal opinion articles, and conceptual or theoretical articles that neither analysed primary nor secondary data. We accounted for all excluded material to appreciate any potential biases or implications of the exclusions to our findings. Table 3 shows the review’s eligibility criteria, including the rationale.

**Table 3:** Studies eligibility criteria

Criteria	Inclusion criteria and rationale	Exclusion criteria and rationale
Population	-Articles reporting on very young adolescents, both female and male (aged 10–14 years). We focused on very young adolescents as programmes mostly leave out this group (28).	-If articles did not report on very young adolescents at all.
Concept	-Articles reporting on social norms/attitudes or perceptions of social norms -Articles reporting on gender norms/attitudes or perceptions of gender norms -Articles reporting on SRH	-Articles not focused on these concepts.
Context	-Studies conducted in any country within SSA.	-Studies conducted outside SSA.
Study design/type of evidence	-Articles reporting findings from primary research. -Articles published between 1 st January 2000 and 31 st December 2024.	-Reviews (e.g., systematic or scoping. reviews) -Articles published before 1 st January 2000.

### Stage 4: data charting

Data charting is the process of synthesising and interpreting data through sorting, charting and organising information based on key issues and themes [38]. In line with Levac et al.’s [42] recommendations, we developed a standard Excel data charting form and populated it with key study characteristics (e.g., author(s), publication year, country/geographical region, study design, study population), and key findings on social and gender norms and SRH. This was an iterative process, involving back-and-forth data extraction and subsequent updating of the data charting form.

We initiated the data charting process with a pilot phase, where two independent reviewers (FAA and ON) charted data from a random sample of 5–10 of the final selected studies. This piloting process determined whether the independent chartings of both reviewers aligned with the review objectives and allowed for any necessary changes to the data charting form. Any disagreements were discussed with a more senior researcher (WM), and the data charting form was revised accordingly.

### Stage 5: collating, summarising and reporting the results

We used descriptive statistics to present the characteristics of the included studies. Specifically, we presented the number of studies meeting study criteria (e.g., geographical location, study period, study design). FAA and ON first carefully reviewed and comprehended the extracted data before developing an initial codebook. Studies were then entered into NVivo 14 (QSR International, Melbourne, Australia) and fully coded using the coding framework; care was taken to identify any additional emerging codes. Codes were grouped and emerging themes were identified. To continuously improve the thematic analysis process, we discussed and shared notes throughout. To better comprehend our findings and pinpoint pertinent gaps in relation to our review questions, we finally merged all of our studies and mapped out the similarities and differences in our data [43]. This final analysis was developed and refined with input from all co-authors.

## Results

### Search outcomes

**Fig. 1:**
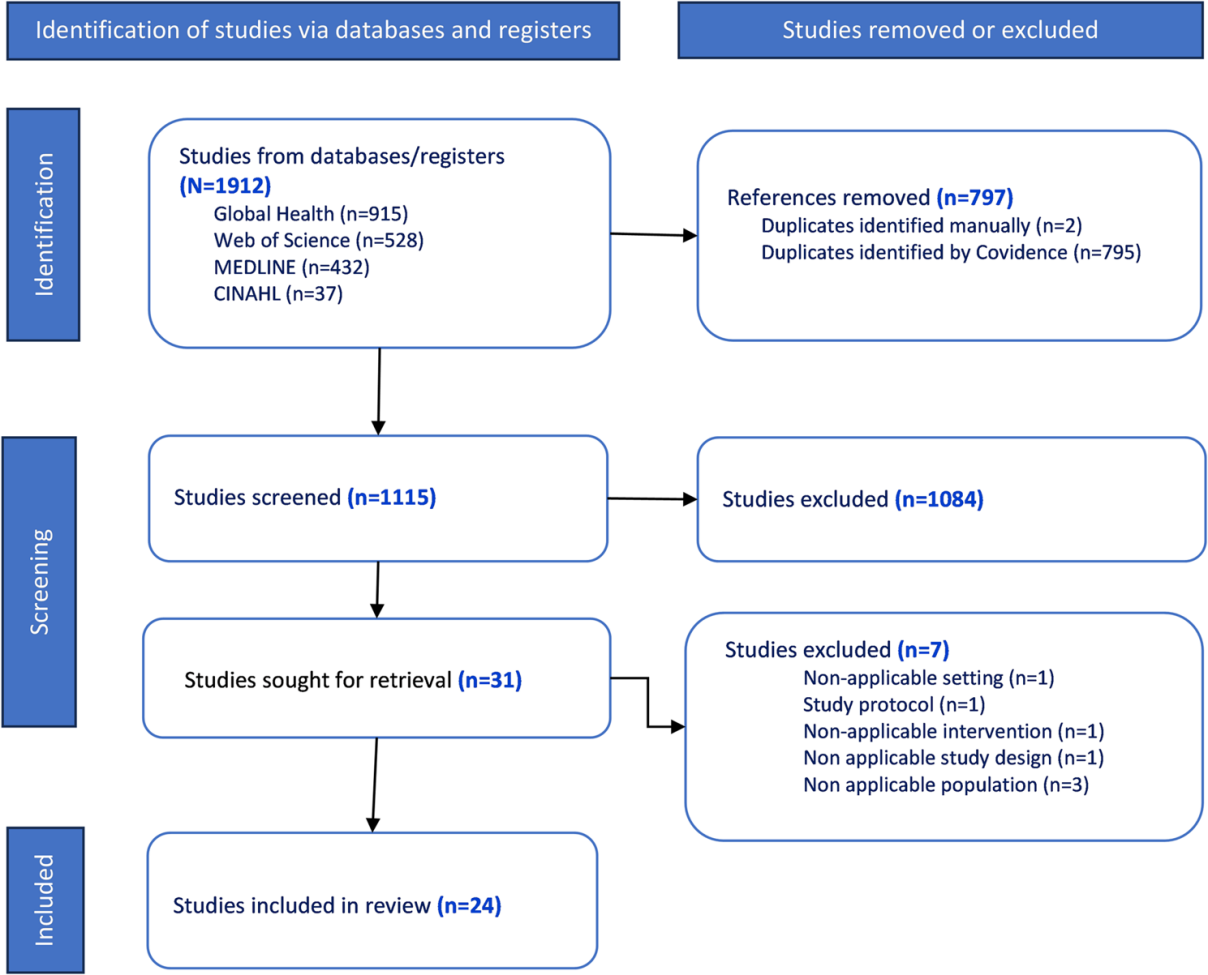
PRISMA flowchart of study selection process

The search yielded 1,912 potentially relevant records from the four databases. Covidence detected 795 duplicate records, and two additional duplicates were removed manually. There remained 1,115 records for title and abstract screening. Title and abstract screening resulted in the exclusion of an additional 1,084 articles, which did not fit the PCC framework components. There remained 31 articles for full-text review. After cross-validation and full-text screening, we included 24 articles in the review. Figure 1 presents a summary of the selection process.

### Study descriptions

The 24 studies included for final review were conducted in nine SSA countries, and distributed across East, South and West Africa, with about two-thirds (*n* = 16, 67%) from East Africa (Table 4). Of the 24 studies, *n* = 7 (29%) were conducted in Uganda, *n* = 6 (25%) in Kenya, with one study [46] in both Kenya and Nigeria. Malawi and the Democratic Republic of the Congo had three studies each. A multi-country study [45] was conducted in Burkina Faso, Ghana, Malawi and Uganda. South Africa and Ethiopia had two studies each, with one of the studies conducted in Ethiopia [64] focusing on Somali refugees. Whilst the review period was 2000–2024, the earliest article was published in 2007. All other papers were published in the last 15 years (i.e., 2009–2024) (Table 4).

**Table 4:** Study characteristics

	Authors	Country	Study design	Study setting	Study population	Key findings on social & gender norms and SRH
1	Baird, S. et al., 2022 [44]	Ethiopia	Mixed methods (RCT & Qualitative)	97 East Hararghe & South Gondar communities	Boys & Girls	• Menstrual stigma and shame; menstruation associated with a curse. • Boys view menstruation as “wrong”, teasing girls during periods. • Sex education perceived as encouraging promiscuity.
2	Bankole, A. et al., 2007 [45]	Burkina Faso, Ghana, Malawi, Uganda	Quantitative	Nationally representative household-based surveys	Boys & Girls	• Very young adolescents already sexually active and many believe their close friends are too. • Very young adolescents had high awareness levels, but little in-depth knowledge of pregnancy and HIV prevention. • Multiple information sources used and preferred.
3	Bello, B.M. et al., 2017 [46]	Kenya, Nigeria	Qualitative	Four urban poor communities in South-western Nigeria & Korogocho slum, Nairobi	Boys & Girls	• Very young adolescents’ reactions to bodily changes vary from anxiety to pride. • Very young adolescents tend to desire greater privacy, trying to hide their developing bodies from others. • Girls emphasise breast development as the marker of puberty, while boys emphasise voice changes. • Among some ethnic groups in Nairobi male circumcision viewed as the hallmark of adolescence.
4	Bunoti, S.N. et al., 2022 [47]	Uganda	Qualitative	16 primary schools in Eastern Uganda	Boys & Girls (in-school)	• Rural girls more aware of their body changes than urban ones. • Urban boys more knowledgeable of pubertal body changes than rural ones. • Girls use herbs as pain killers to reduce menstrual pain. • Poor pubertal hygiene practices such as shaving due to limited knowledge and resources. • Poor parent-child communication related to sex and puberty.
5	Chimwaza-Manda, W. et al., 2021 [48]	Malawi	Qualitative	Girls-only club participants in two rural southern districts	Girls	• Due to gender norms, very young adolescent girls have fewer opportunities than boys to socialise and engage in leisure and income-generating activities. • Gender differences where household chores done by girls and boys left to play.
6	Chimwaza-Manda, W. et al., 2023 [49]	Malawi	Qualitative	Girls-only club participants in two rural southern districts	Girls	• Parents and adult relatives often advise against engaging in sexual relationships to prevent pregnancy, preventing some Very young adolescent girls from discussing SRH issues. • Very young adolescents rely on peers’ incorrect information.
7	Chimwaza-Manda, W. et al., 2024 [50]	Malawi	Qualitative	Girls-only club participants in two rural southern districts	Girls	• Very young adolescent girls’ social dynamics are influenced by their trust and confidence in SRH information sources. • Parents/caregivers only start to talk to very young adolescent girls about SRH issues (e.g., prevention of pregnancy) when they have already started menstruating.
8	Cislaghi, B. et al., 2021 [51]	DRC	Quantitative	Two largest urban poor communes of Kinshasa	Boys & Girls (in-school and out-of-school)	• Changes in Sexual Double Standard (SDS) scores are linked to puberty and pubertal onset, with girls facing increased pressure to maintain their sexual purity. • Boys reported greater freedom of movement, spent more time with peers, and socialised in mixed-sex groups.
9	Gayles, J. et al., 2023 [52]	DRC	Mixed methods (Quantitative & Qualitative)	Two largest urban poor communes of Kinshasa	Boys & Girls (in-school and out-of-school)	• Discriminatory attitudes towards gender nonconforming adolescents. • Unequal gender norms, such as household chore sharing.
10	Kagesten, A.E. et al., 2018 [53]	Kenya	Quantitative	Korogocho slum, Nairobi	Boys & Girls	• Parents disapprove of sexual relationships in very young adolescents for both boys and girls. • Masculine norms become stereotypical in early adolescence.
11	Kemigisha, E. et al., 2018a [54]	Uganda	Quantitative	33 urban & rural primary schools in Mbarara district	Boys & Girls (in-school)	• SRH knowledge among Very young adolescents is poor. • There are significant gaps in information about early sexual debut and risky practices.
12	Kemigisha, E. et al., 2018b [55]	Uganda	Quantitative	33 urban & rural primary schools in Mbarara district	Boys & Girls	• Very young adolescents socialised at a young age to have inequitable norms • Boys perceived as cleverer, and girls expected to stay home. • Girls had higher gender equitable norm scores.
13	Kemigisha, E. et al., 2019 [56]	Uganda	Mixed methods (RCT & Qualitative)	33 urban & rural primary schools in Mbarara district	Boys & Girls	• Sexual wellbeing and attitudes based on self-esteem and body image and gender equitable norms.
14	Maina, B. et al., 2020a [57]	Kenya	Quantitative	Korogocho slum, Nairobi	Boys	• Young people begin being romantically involved at an early age. • Very young adolescents understand social norms and power dynamics in heteronormative romantic relationships, but they do not apply these beliefs to their sexual activities. • Boys’ endorsement of heteronormativity in romantic relationships was low, suggesting they disagreed with traditional gender judgments o However, their endorsement of male dominance in romantic relationships had a significant inverse association with sexual experiences.
15	Maina, B. et al., 2020b [58]	Kenya	Quantitative	Korogocho and Viwandani slums, Nairobi	Girls	• Girls who had experienced violence were more likely to be sexually experienced. • Poor mental health was reportedly associated with risky sexual behaviours.
16	Maina, B. et al., 2020c [59]	Kenya	Qualitative	Korogocho slum, Nairobi	Boys & Girls	• Parents discourage romantic relationships among very young adolescents, viewing them as an obstacle to educational achievement. • Lack of communication about sexual relationships is primarily due to the perception that children are too young to engage in romantic relationships. • Fear-based communication, including threats, is employed to prevent romantic relationships. • Discussing sex-related matters with adolescents may be interpreted as approval or tolerance of promiscuity.
17	Maina, B. et al., 2022 [60]	Kenya	Qualitative	Korogocho slum, Nairobi	Boys	• Boys observe older men’s behaviours and roles, receive messages on masculine expectations from parents and teachers. • Contextual masculinity is associated with circumcision, and Muslim boys perceive masculinity based on religious teachings. • Media and internet messages portray men as expected to support and take family financial responsibilities.
18	Mda, P. et al., 2013 [61]	South Africa	Qualitative	Two urban primary schools in Eastern Cape	Girls (in-school)	• Parents fear discussing contraceptives and romantic relationships with very young adolescent girls. • Older girls responsible for teaching young girls once they reach puberty. • Adolescent pregnancy rates are linked to ignorance about contraception, coercion, rape and low modern contraceptives access.
19	Ninsiima, A.B. et al., 2018 [62]	Uganda	Qualitative	Six urban and rural primary schools in Mbarara district	Boys & Girls (in-school)	• Long-term gender ideals include girls’ values for motherhood, marriage and caregiving. • Social norms require parents and teachers to control girls more than boys. • Girls are expected to control their own sexuality and take responsibility for boys’ sexualities; prevention of pregnancy is perceived as a girl’s responsibility. • Boys are generally independent and have unlimited mobility, while girls are socially expected to be submissive and have restricted movements. • Sexuality education mainly focuses on biological aspects like hygiene and control of girls’ sexuality. • Gender norms form early in life and create unequal power relations that constrain adolescents from exercising agency.
20	Nyakato, V.N. et al., 2021 [63]	Uganda	Participatory	Southwestern rural Uganda	Boys & Girls (in-school)	• When a girl gets pregnant, it brings shame to the family and more particularly, the mother and other girls. • Traditional cultural norms emphasise gender differences, with boys working on farms, plantations, and gardens, while girls maintain the household and care for younger siblings.
21	Ortiz-Echevarria, L. et al., 2017 [64]	Somali refugees in Ethiopia	Qualitative	Kobe refugee camp	Boys & Girls	• Inequitable relations between boys and girls, and harmful traditional practices speak to the lack of self-efficacy and decision-making that very young adolescents and specifically girls, experience. • Girls faced an additional risk of child marriage and early pregnancy, with displacement significantly limiting their ability to access education and achieve future aspirations.
22	Selikow, T.A. et al., 2009 [65]	South Africa	Qualitative	Cape Town metropolis schools	Boys & Girls (in-school)	• Limited access to adults‚ knowledge and hence the adolescents rely on peers for information. • Adolescents often feel a sense of belonging in groups, which can be exploited to promote negative sexual norms. • Peer pressure among both boys and girls undermines healthy social norms and HIV prevention messages to abstain, be faithful, use a condom and delay sexual debut.
23	Tchuisseu et al., 2023 [66]	Uganda	Mixed methods (participatory & FGDs)	Rural community outside Mbarara, Uganda	Boys & Girls	• Girls are expected to avoid pregnancies. • Girls receive more warnings and stay at home, while boys are not always expected to stay at home. • Limited economic resources, sex education and gender expectations can lead to negative SRH outcomes. • School and peers provide sex education, but girls are more likely to experience rape and transactional sex.
24	Zimmerman, L.A. et al., 2021 [67]	DRC	Quantitative	Two largest urban poor communes of Kinshasa	Boys & Girls (in-school and out-of-school)	• An increase in sexual double standards among adolescents who have not yet reached puberty. • While no significant sex differences exist in decision-making power, boys have greater voice and representation.

### Study characteristics

Of the 24 studies, *n* = 11 (46%) were exclusively qualitative [46–50, 57, 60–62, 64, 65] and *n* = 8 (33%) were entirely quantitative [45, 51, 53–55, 58, 59, 67]. Three other quantitative studies [44, 52, 56] including two cluster-randomised trials [44, 56] incorporated qualitative components. One study [63] exclusively used participatory techniques while the other [66] used these alongside focus group discussions. All studies included large, representative samples, appropriate methodologies, and were rigorously conducted. Studies were conducted in poor rural and urban settings, including slums. Seven (29%) studies [47, 54–56, 61, 62, 65] specifically targeted in-school boys and girls.

Overall, *n* = 17 (71%) studies explored social norms and SRH among both boys and girls, *n* = 5 (21%) among only girls and *n* = 2 (8%) among only boys (Table 4). Four (17%) studies studied very young adolescents alongside other population groups including older adolescents [64], parents/caregivers [44, 57, 62], teachers [62] and other community influencers [44, 64]. One study investigated very young adolescents’ SRH experiences from the perspective of emerging adults (18–25 years old) [66].

Six (25%) studies [44, 48–50, 52, 56] evaluated the effectiveness of norms-shifting interventions targeting very young adolescents. Two [44, 52] specifically evaluated the effectiveness of gender-transformative interventions. Of these, one assessed the impact of a gender-transformative life-skills intervention (Act With Her-Ethiopia [AWH-E]) on the menstrual health literacy of very young adolescent girls and boys [44]. Another evaluated the impact of Growing Up GREAT! on SRH knowledge, assets and agency, and gender-equitable attitudes and behaviours among VYA participants [52]. A third study evaluated the effectiveness of a comprehensive SRH intervention for very young adolescents implemented in 33 primary schools [56]. Three other studies explored the impact of girl-only clubs that were part of a larger comprehensive HIV prevention project - DREAMS (Determined, Resilient, Empowered, AIDS-free, Mentored, and Safe) [48–50]. DREAMS interventions included improved access to key health services, education support, social skills, asset building, and economic strengthening [24, 48, 68]. Key reported issues are summarised in Table 4 and described in detail below.

### Key reported issues

#### Beliefs, practices and very young adolescents’ SRH

The review identified several norms linked to very young adolescents’ SRH, including menstrual-related norms and associated stigma, shame and limited mobility [44]. Adult females only discussed menstruation with very young adolescents after it had commenced. Additionally, discussions rarely focused on SRH-related aspects but rather, on the need to maintain hygiene and to use traditional remedies to manage associated pain (e.g., herbs) [47]. In some settings, male circumcision was associated with masculinity when uncircumcised boys were both looked down upon and ridiculed, with some eventually getting circumcised to both gain social acceptance and avoid stigmatisation [60]. A study conducted in South Africa illustrated how hegemonic traditional masculine norms sometimes led to negative sexual norms when non-sexually experienced very young adolescent boys were labelled *“umqwayito”* (dried fruit or meat) – derogatory terms which forced some to engage in (sometimes risky) sexual activity to conform to group norms [65].

#### Parental communication and gatekeeping

Overall, parents, caregivers and other adults believed that very young adolescents were too young to engage in romantic relationships and therefore, avoided discussing SRH issues with them [49, 59]. Consequently, very young adolescents had to rely on their peers and the media for SRH information [65], but often found it challenging to determine the information’s accuracy [60, 63]. They were therefore prone to myths and misconceptions. For example, in one study, very young adolescents reported fear of using contraceptive pills due to perceptions that they have long-term effects, including infertility [61].

Additionally, some parents perceived any romantic interactions during very young adolescence as a hindrance to academic achievement [59]. Others regarded such relationships as disgraceful to the family or generally immoral. Consequently, parents used threats to deter such relationships, which in turn, resulted in very young adolescents feeling scared to either discuss or disclose their feelings and experiences, leading to a breakdown in healthy communication [57].

There was evidence of differential treatment between very young adolescent boys and girls during the pubertal stage when carers became more concerned about the girls’ sexuality. This often led to heightened expectations for very young adolescent girls to behave in a respectable manner and maintain their sexual purity to prevent bringing shame upon their families [51]. This was compounded by the general fear that girls who fall pregnant may not be accepted by the perpetrator’s family [63]. Of note, girls were expected to simultaneously exert social control over their own and boys’ sexuality due to the perception that pregnancy is primarily a girl’s responsibility [63].

#### Double standards relating to very young adolescents’ SRH

Due to concerns around very young adolescent girls’ heightened vulnerability, parents and other stakeholders (e.g., teachers) instituted stricter measures on very young adolescent girls compared to boys [62]. For example, study participants mentioned that very young adolescent boys enjoyed free movement and could have multiple, concurrent partners while girls’ sexuality was restricted [53, 62]. Girls who expressed sexual agency were perceived as abnormal or promiscuous by boys and adults [62]. Boys maintained that in heterosexual romantic relationships, they were supposed to be dominant, with girls expected to be passive [53, 67].

Two studies [51, 67] explored a specific gender norm: the Sexual Double Standard (SDS), a measure of different normative expectations for romantic activities, rewarding boys but devaluating girls for engaging in the same behaviours [28]. The studies found a high SDS prevalence among very young adolescents, which increased over time, and was influenced by pubertal onset, family interactions, peer interactions and media exposure [51, 67]. In one of the studies, very young adolescent boys agreed that boys and girls should face different judgements for the same sexual behaviour [51].

#### Contextual factors

From the review, it was evident that contextual factors have a bearing on very young adolescents’ SRH-related attitudes, behaviours and ultimately outcomes. For example, living in urban informal settlements led to comparatively higher common mental disorders, including anxiety and depression as these settings exhibit significant social, environmental and physical risks [53]. These mental health challenges increased the risk of engaging in risky sexual behaviours [58]. Studies also demonstrated that factors at various level impact very young adolescents’ SRH. For example, household dynamics, including parental loss, likely lead to early sexual debut [50]. At the community level, sociocultural factors including ethnicity, cultural practices, gender roles, cultural beliefs and safety impact very young adolescents’ SRH-related attitudes, behaviours and ultimately, outcomes [64, 67].

#### Impact of interventions

Evaluations of interventions targeting social and gender norms relating to SRH-related attitudes and behaviours produced mixed results. For example, a trial testing effectiveness of the AWH-E intervention on the menstrual health literacy of very young adolescent boys and girls found statistically significant improvements on norms around menstruation, knowledge about menstruation and biological function, and knowledge and behaviour related to menstrual hygiene management, but with important differences by gender and location [44]. The Growing Up GREAT! gender-transformative initiative successfully improved SRH knowledge, assets, and gender-equitable attitudes and behaviours, but did not shift gender equitable norms [52]. Similarly, a school-based intervention improved SRH knowledge among intervention schools, but found no significant differences in self-esteem, body image or gender equitable norms [56]. Overall, intervention studies highlighted the importance of adapting gender-responsive/gender-transformative interventions to the local context.

## Discussion

This review explored how social and gender norms influence the sexual and reproductive health (SRH)-related attitudes and behaviours of very young adolescents (10–14 years) in Sub-Saharan Africa (SSA). All but one of the studies included in the review were conducted in the last 15 years, reflecting an increasing but comparatively nascent interest in this age group. Identified norms include those relating to menstruation, puberty, circumcision, romantic relationships, and gender stereotypes. We treated gender norms as a subset of social norms as the operational distinction between the two concepts became blurred in the review results (i.e., some norms could be categorised as social/gender norms).

Overall, norms restrict very young adolescent girls’ access to SRH information and services. A recurring theme is that parents, teachers and services providers continue to believe that very young adolescents are too young to be sexually active or to need SRH information [30–32]. Very young adolescents therefore end up relying on the media and their peers for SRH information [65]. Peers do not often have adequate or correct information; this is also true for older adolescents. A study among girls aged 15–19 years in Uganda [69] found that even these older adolescents have challenges accessing SRH information. This highlights a common gap among adolescents regardless of their age, and the need to provide this group with adequate, correct SRH information. One of the reasons for the gap could be because very young adolescents are often seen as children and older adolescents as adults [33]. There is need to recognise adolescents as a distinct group, with own specific needs and issues, and provide appropriate SRH information.

The review identified parental challenges around discussing SRH issues with very young adolescents, even if they wanted to do so. Without adequate knowledge and skills, the communication predominantly involves conveying warnings, issuing threats and resorting to physical discipline [57]. However, recent research on the impact of parenting on adolescent sexual risk-taking has demonstrated that supportive parent-child relationships can reduce the risk of adolescents engaging in unprotected sexual activities [70]. Interventions targeting very young adolescents’ SRH should therefore include a component focusing on parent-child relationships.

Among very young adolescent boys, both peer pressure and the need to conform to group norms are linked to SRH behaviours and practices. Of concern, however, is the influence of hegemonic traditional masculine norms which sometimes result in very young adolescent boys engaging in aggressive sexual activity [65]. Of note, in some reviewed studies, very young adolescent girls reported sexual coercion and rape [61, 66]. Adolescents in general and very young adolescents, in particular assign greater weight than adults to social outcomes such as peer acceptance [71]. Previous research has characterised the development of vulnerability to peer influence during adolescence as a pattern that resembles an inverted U-shaped curve, showing an initial rise during early adolescence, reaching its highest point around 14 years of age, and thereafter decreasing [72]. Very young adolescents therefore exhibit lower levels of resistance to peer pressure compared to both children and adults [72]. Interventions focusing on very young adolescents should therefore include life skills to deal with peer pressure.

On the whole, however, very young adolescents’ SRH is influenced by a complex interplay of social, cultural, and economic factors. For example, among adolescents in impoverished areas (e.g., slums), very young adolescents’ SRH is influenced by numerous intersectional factors such as crime, prostitution, violence and substance use [17, 59]. These factors may shape for example, very young adolescents’ masculinities but importantly, affect their mental well-being. Reviewed studies suggested a link between poor mental health and SRH outcomes. There is a recognised bidirectional relationship between the two in that poor mental health leads to poor SRH outcomes and vice versa [73]. Further, depression, HIV and self-harm are three of the top five global causes of disability-adjusted life years lost for adolescents [74]. Interventions need to recognise these linkages and avoid the tendency to focus on just one aspect and not the other. Above all, given the various intersecting factors, interventions need to be careful not to over-emphasise individual determinants of health, overlooking other factors that shape life chances, health risks, and vulnerabilities [75]. Finally, and as found by intervention studies, context is key and context-specific adaptations are critical [44].

### Gaps in the literature and recommendations for future programming and research

A clear gap in the literature was the lack of focus on emerging settings in which social and gender norms are produced and reproduced such as social media. With the growing number of mobile phone users in SSA, mobile health (mHealth) – promotion of health-related issues through mobile phones, tablets and other digital devices – programming and research should continue to explore the devices’ potential to influence norms related to very young adolescents’ access to and use of SRH services [76].

Reviewed studies focused on some groups of very young adolescents often left out by both programming and research (e.g. slum dwellers, refugees), which is commendable. Future programming and research should, however, focus on other groups with limited access to SRH services and interventions, including those living on the streets, in servitude and, gender non-conforming adolescents. Finally, only a few studies (17%) studied both very young adolescents and their influential others. In keeping with the socioecological model, which shows how individuals are embedded within larger social systems [77], interventions need to also focus on very young adolescents’ wider support system. It will also be important to identify and explore the community resources and structures available to ensure interventions’ sustainability and scalability.

### Strengths and limitations

This scoping review allowed us to broadly examine how social norms influence the SRH of very young adolescents in SSA. The strength of the review is in the application of a recognised, thorough and transparent approach [38] to review the literature and report our results. We also employed a two-stage, double screening process to minimise bias. However, given that a significant part of SSA is Lusophone or Francophone, and programmes in these regions are disproportionately funded [4], this likely skewed study results. Further, 67% of studies were from three East African countries (Ethiopia, Kenya, Uganda), reflecting disproportionate geographical distribution. Finally, we excluded grey literature; relevant evidence from community-based or NGO-led initiatives may therefore have been missed.

## Conclusions

Scoping review findings affirm the importance of intervening in very young adolescence to influence positive social and gender norms. They highlight the importance of engaging parents, caregivers and other influential adults as key influencers of very young adolescents’ attitudes, behaviours, and norms related to SRH [52]. The review also highlights the need for multifaceted, culturally sensitive, gender-responsive interventions to improve very young adolescents’ SRH-related attitudes and behaviours in Sub-Saharan Africa.

## Supplementary Information

Supplementary Material 1.

Supplementary Material 2.

## Data Availability

All data generated or analysed during this study are included in this published article.

## Abbreviations

AWH-E: Act With Her-Ethiopia
CINAHL: Cumulative Index to Nursing and Allied Health Literature
DREAMS: Determined, Resilient, Empowered, AIDS-free, Mentored, and Safe
GEAS: Global Early Adolescent Study
HIV: Human Immunodeficiency Virus
MEDLINE: Medical Literature Analysis and Retrieval System Online
mHealth: Mobile health
NGO: Non-Governmental Organisation
OFS: Open Science Framework
PPC: Population–Concept–Context
PRISMA-ScR: Preferred Reporting Items for Systematic Reviews and Meta‐Analyses Extension for Scoping Reviews
PROSPERO: Prospective Register Of Systematic Reviews
SDS: Sexual Double Standards
SRH: Sexual and reproductive health
SSA: Sub-Saharan Africa
WHO: World Health Organisation

## References

[1] WHO. Adolescent Health. Available from: https://www.who.int/southeastasia/health-topics/adolescent-health. Accessed 20 2025.

[2] Shinde S, Harling G, Assefa N, Barnighausen T, Bukenya J, Chukwu A, et al. Counting adolescents in: the development of an adolescent health indicator framework for population-based settings. EClinicalMedicine. 2023;61:102067. 37448809 10.1016/j.eclinm.2023.102067 PMC10336247

[3] Canning D, Raja S, Yazbeck A. Africa’s Demographic Transition : Dividend or Disaster? Washington DC: World Bank; 2015.

[4] Ahmed F, Nyamwanza O, Ladur AN, Dambi J, Cowan F, Mavhu W. How do social norms influence the sexual and reproductive health of very young adolescents in Sub-Saharan africa? A scoping review protocol. Wellcome Open Res. 2024;9:670. 40070665 10.12688/wellcomeopenres.23139.2 PMC11894366

[5] Sedgh G, Bearak J, Singh S, Bankole A, Popinchalk A, Ganatra B, et al. Abortion incidence between 1990 and 2014: global, regional, and subregional levels and trends. Lancet. 2016;388(10041):258–67. 27179755 10.1016/S0140-6736(16)30380-4 PMC5498988

[6] Munakampe MN, Zulu JM, Michelo C. Contraception and abortion knowledge, attitudes and practices among adolescents from low and middle-income countries: a systematic review. BMC Health Serv Res. 2018;18(1):909. 30497464 10.1186/s12913-018-3722-5 PMC6267062

[7] Maharaj NR. Adolescent pregnancy in Sub-Saharan Africa - a cause for concern. Front Reprod Health. 2022;4:984303. 36531444 10.3389/frph.2022.984303 PMC9755883

[8] WHO. Report on global sexually transmitted infection surveillance. Geneva: World Health Organization; 2018.

[9] Hatzold K, Gudukeya S, Mutseta MN, Chilongosi R, Nalubamba M, Nkhoma C, et al. HIV self-testing: breaking the barriers to uptake of testing among men and adolescents in Sub-Saharan Africa, experiences from STAR demonstration projects in Malawi, Zambia and Zimbabwe. J Int AIDS Soc. 2019;22(1):e25244. 30907505 10.1002/jia2.25244 PMC6432104

[10] Brink DT, Martin-Hughes R, Bowring AL, Wulan N, Burke K, Tidhar T, et al. Impact of an international HIV funding crisis on HIV infections and mortality in low-income and middle-income countries: a modelling study. Lancet HIV. 2025;12(5):e346–54. 40157378 10.1016/S2352-3018(25)00074-8

[11] Sibanda EL, Phillips AN. Modelling study shows staggering impact of HIV funding cuts. Lancet HIV. 2025;12(5):e316–8. 40157375 10.1016/S2352-3018(25)00076-1

[12] Sidibe M, Makgoba TC, Paul B, Brilliant B, Banda JHK, Sichone Cameron M, et al. Accelerating domestic investments to end AIDS in Africa. Lancet. 2025;405(10487):1335–6. 40215989 10.1016/S0140-6736(25)00685-3

[13] Murewanhema G, Musuka G, Moyo P, Moyo E, Dzinamarira T. HIV and adolescent girls and young women in Sub-Saharan Africa: a call for expedited action to reduce new infections. IJID Reg. 2022;5:30–2. 36147901 10.1016/j.ijregi.2022.08.009 PMC9485902

[14] Maina BW, Izugbara C, Sikweyiya Y, Nandongwa I, Kabiru CW. Gender Norms And Sexual Behavior Among Very Young Adolescents In Sub-Saharan Africa: A scoping review. In: The Routledge Handbook of African Demography. London: Routledge; 2022. p. 330 – 48.

[15] Melesse DY, Mutua MK, Choudhury A, Wado YD, Faye CM, Neal S, et al. Adolescent sexual and reproductive health in Sub-Saharan africa: who is left behind? BMJ Glob Health. 2020;5(1):e002231. 32133182 10.1136/bmjgh-2019-002231 PMC7042602

[16] Cislaghi B, Heise L. Gender norms and social norms: differences, similarities and why they matter in prevention science. Sociol Health Illn. 2020;42(2):407–22. 31833073 10.1111/1467-9566.13008 PMC7028109

[17] Nyamwanza O, Bikwayi TS, Chinozvina T, Makoni L, Muronzi F, Changombe M, et al. Exploring gender stereotypes and norms among peri-urban very young adolescents in Zimbabwe using participatory and qualitative approaches. PLOS Glob Public Health. 2025;5(5):e0003845. 40440317 10.1371/journal.pgph.0003845 PMC12121727

[18] Doyle K, Levtov RG, Barker G, Bastian GG, Bingenheimer JB, Kazimbaya S, et al. Gender-transformative Bandebereho couples’ intervention to promote male engagement in reproductive and maternal health and violence prevention in Rwanda: findings from a randomized controlled trial. PLoS One. 2018;13(4):e0192756. 29617375 10.1371/journal.pone.0192756 PMC5884496

[19] Doyle K, Bhatnagar I, Karamage E, Tuyisingize JP, Muhimpundu C, Nyiransabimana AMY, et al. Equipping community health workers in Rwanda to deliver a gender transformative parenting program to prevent violence against women and children at scale. Front Reprod Health. 2025;7:1602136. 10.3389/frph.2025.1602136.eCollection 2025. 10.3389/frph.2025.1602136 PMC12230087 40625533

[20] Wanders F, Homan AC, van Vianen AEM, Rahal RM, van Kleef GA. How norm violators rise and fall in the eyes of others: the role of sanctions. PLoS One. 2021;16(7):e0254574. 34324549 10.1371/journal.pone.0254574 PMC8324048

[21] Mavhu W, Langhaug L, Pascoe S, Dirawo J, Hart G, Cowan F. A novel tool to assess community norms and attitudes to multiple and concurrent sexual partnering in rural zimbabwe: participatory attitudinal ranking. AIDS Care. 2011;23(1):52–9. 21218276 10.1080/09540121.2010.490257

[22] Ampofo AA. When men speak women listen: gender socialisation and young adolescents’ attitudes to sexual and reproductive issues. Afr J Reprod Health. 2001;5(3):196–212. 12471941

[23] Mavhu W, Langhaug L, Manyonga B, Power R, Cowan F. What is ‘sex’ exactly? Using cognitive interviewing to improve the validity of sexual behaviour reporting among young people in rural Zimbabwe. Cult Health Sex. 2008;10(6):563–72. 18649195 10.1080/13691050801948102

[24] Mavhu W, Rowley E, Thior I, Kruse-Levy N, Mugurungi O, Ncube G, et al. Sexual behavior experiences and characteristics of male-female partnerships among HIV positive adolescent girls and young women: qualitative findings from Zimbabwe. PLoS One. 2018;13(3):e0194732. 29566062 10.1371/journal.pone.0194732 PMC5864257

[25] Amin A, Kagesten A, Adebayo E, Chandra-Mouli V. Addressing gender socialization and masculinity norms among adolescent boys: policy and programmatic implications. J Adolesc Health. 2018;62(3S):S3–5. 29455715 10.1016/j.jadohealth.2017.06.022 PMC5817048

[26] Editor. The lost boys. Lancet Child Adolesc Health. 2019;3(7):437. 31155320 10.1016/S2352-4642(19)30154-3

[27] Marcus R, Stavropoulou M. N. A-G. Programming with adolescent boys to promote gender-equitable masculinities: a rigorous review. London: GAGE; 2018.

[28] Moreau C, Li M, De Meyer S, Vu Manh L, Guiella G, Acharya R, et al. Measuring gender norms about relationships in early adolescence: results from the global early adolescent study. SSM Popul Health. 2019;7:014–14. 30581959 10.1016/j.ssmph.2018.10.014 PMC6293033

[29] Duell N, Steinberg L, Icenogle G, Chein J, Chaudhary N, Di Giunta L, et al. Age patterns in risk taking across the world. J Youth Adolesc. 2018;47(5):1052–72. 29047004 10.1007/s10964-017-0752-y PMC5878702

[30] Kaufman MR, Dam KH, Van Lith LM, Hatzold K, Mavhu W, Kahabuka C, et al. Voluntary medical male circumcision among adolescents: a missed opportunity for HIV behavioral interventions. AIDS. 2017;31(Suppl 3):S233–41. 28665881 10.1097/QAD.0000000000001484 PMC5497778

[31] Kaufman MR, Patel EU, Dam KH, Packman ZR, Van Lith LM, Hatzold K, et al. Counseling received by adolescents undergoing voluntary medical male circumcision: moving toward Age-Equitable comprehensive human immunodeficiency virus prevention measures. Clin Infect Dis. 2018;66(suppl3):S213–20. 29617776 10.1093/cid/cix952 PMC5889033

[32] Tobian AAR, Dam KH, Van Lith LM, Hatzold K, Marcell AV, Mavhu W, et al. Providers’ perceptions and training needs for counseling adolescents undergoing voluntary medical male circumcision. Clin Infect Dis. 2018;66(suppl3):S198–204. 29617772 10.1093/cid/cix1036 PMC5888966

[33] Mmari K, Simon C, Verma R. Gender-transformative interventions for young adolescents: what have we learned and where should we go? J Adolesc Health. 2024;75(4S):S62-80. 39293879 10.1016/j.jadohealth.2024.04.016

[34] Mmari K, Blum RW, Atnafou R, Chilet E, de Meyer S, El-Gibaly O, et al. Exploration of gender norms and socialization among early adolescents: the use of qualitative methods for the global early adolescent study. J Adolesc Health. 2017;61(4S):S12–8. 28915986 10.1016/j.jadohealth.2017.07.006

[35] Mmari K. Are gender-transformative interventions effective among very young adolescents? J Adolesc Health. 2023;73(1S):S1-2. 37330816 10.1016/j.jadohealth.2023.03.010

[36] Rwabyoma A, Muriisa R, Ochieng D, Rubagiza J. Men and Masculinity Studies in East Africa: A Theoretical Framework for Understanding Masculinities-Focused Interventions. In: he Palgrave Handbook of African Men and Masculinities. London: Palgrave Macmillan; 2024.

[37] Tricco AC, Lillie E, Zarin W, O’Brien KK, Colquhoun H, Levac D, et al. PRISMA extension for scoping reviews (PRISMA-ScR): checklist and explanation. Ann Intern Med. 2018;169(7):467–73. 30178033 10.7326/M18-0850

[38] Arksey H, O’Malley L. Scoping studies: towards a methodological framework. Int J Soc Res Methodol. 2005;8(1):19–32.

[39] Pollock D, Peters MDJ, Khalil H, McInerney P, Alexander L, Tricco AC, et al. Recommendations for the extraction, analysis, and presentation of results in scoping reviews. JBI Evid Synthesis. 2023;21(3):520–32. 10.11124/JBIES-22-00123 36081365

[40] Ajayi AI, Otukpa EO, Mwoka M, Kabiru CW, Ushie BA. Adolescent sexual and reproductive health research in Sub-Saharan africa: a scoping review of substantive focus, research volume, geographic distribution and Africa-led inquiry. BMJ Glob Health. 2021;6(2): e004129. 10.1136/bmjgh-2020-004129. 10.1136/bmjgh-2020-004129 PMC7878134 33568395

[41] United Nations. Transforming our world: The 2030 agenda for sustainable development. New York: United Nations, Department of Economic and Social Affairs.; 2015.

[42] Levac D, Colquhoun H, O’Brien KK. Scoping studies: advancing the methodology. Implement Sci. 2010;5(1):69. 20854677 10.1186/1748-5908-5-69 PMC2954944

[43] Kraus S, Breier M, Lim WM, Dabić M, Kumar S, Kanbach D, et al. Literature reviews as independent studies: guidelines for academic practice. RMS. 2022;16(8):2577–95.

[44] Baird S, Hamory J, Gezahegne K, Pincock K, Woldehanna T, Yadete W, et al. Improving menstrual health literacy through life-skills programming in rural Ethiopia. Front Glob Womens Health. 2022;3:838961. 35873135 10.3389/fgwh.2022.838961 PMC9304804

[45] Bankole A, Biddlecom A, Guiella G, Singh S, Zulu E. Sexual behavior, knowledge and information sources of very young adolescents in four Sub-Saharan African countries. Afr J Reprod Health. 2007;11(3):28–43. 18458739 PMC2367131

[46] Bello BM, Fatusi AO, Adepoju OE, Maina BW, Kabiru CW, Sommer M, et al. Adolescent and parental reactions to puberty in Nigeria and kenya: A Cross-Cultural and intergenerational comparison. J Adolesc Health. 2017;61(4S):S35–41. 28915991 10.1016/j.jadohealth.2017.03.014

[47] Bunoti SN, Tumwesigye NM, Atuyambe L. Awareness of pubertal body changes among primary school children aged 10–14 years in Eastern Uganda; challenges and opportunities. Reprod Health. 2022;19(1):180. 35986331 10.1186/s12978-022-01466-y PMC9392265

[48] Chimwaza-Manda W, Pilgrim N, Kamndaya M, Mathur S, Sikweyiya Y. Girl-only clubs’ influence on SRH knowledge, HIV risk reduction, and negative SRH outcomes among very young adolescent girls in rural Malawi. BMC Public Health. 2021;21(1):806. 33906614 10.1186/s12889-021-10874-x PMC8077750

[49] Chimwaza-Manda W, Kamndaya M, Pilgrim N, Mathur S, Chipeta EK, Sikweyiya Y. Social support and very young adolescent girl’s knowledge on sexual relationships: a comparative qualitative study of Girl only clubs’ participants and non-participants in rural Malawi. PLOS Glob Public Health. 2023;3(1):e0001339. 36962900 10.1371/journal.pgph.0001339 PMC10022037

[50] Chimwaza-Manda W, Kamndaya M, Chipeta EK, Sikweyiya Y. Sexual health knowledge acquisition processes among very young adolescent girls in rural malawi: implications for sexual and reproductive health programs. PLoS One. 2024;19(2):e0276416. 38394159 10.1371/journal.pone.0276416 PMC10889655

[51] Cislaghi B, Bhatia A, Li M, Lian Q, Baird S, Kayembe P, et al. Changes in the sexual double standard associated with sociodevelopmental factors among young adolescents in Kinshasa. J Adolesc Health. 2021;69(1S):S23–30. 34217455 10.1016/j.jadohealth.2020.07.041

[52] Gayles J, Yahner M, Barker KM, Moreau C, Li M, Koenig L, et al. Balancing quality, intensity and scalability: results of a multi-level sexual and reproductive health intervention for very young adolescents in Kinshasa. J Adolesc Health. 2023;73(1S):S33–42. 37330819 10.1016/j.jadohealth.2023.02.001

[53] Kagesten AE, Kabiru CW, Maina B, German D, Blum RW. Inexperienced’? Patterns in romantic and sexual experiences among urban poor early adolescents in Nairobi, Kenya. Cult Health Sex. 2018;20(12):1299–316. 29558253 10.1080/13691058.2018.1432765

[54] Kemigisha E, Bruce K, Nyakato VN, Ruzaaza GN, Ninsiima AB, Mlahagwa W, et al. Sexual health of very young adolescents in South Western Uganda: a cross-sectional assessment of sexual knowledge and behavior. Reprod Health. 2018;15(1):148. 30157881 10.1186/s12978-018-0595-3 PMC6114035

[55] Kemigisha E, Nyakato VN, Bruce K, Ndaruhutse Ruzaaza G, Mlahagwa W, Ninsiima AB, et al. Adolescents’ sexual wellbeing in southwestern Uganda: a cross-sectional assessment of body image, self-esteem and gender equitable norms. Int J Environ Res Public Health. 2018;15(2):372. 10.3390/ijerph15020372. 10.3390/ijerph15020372 PMC5858441 29470388

[56] Kemigisha E, Bruce K, Ivanova O, Leye E, Coene G, Ruzaaza GN, et al. Evaluation of a school based comprehensive sexuality education program among very young adolescents in rural Uganda. BMC Public Health. 2019;19(1):1393. 31660918 10.1186/s12889-019-7805-y PMC6819440

[57] Maina BW, Ushie BA, Kabiru CW. Parent-child sexual and reproductive health communication among very young adolescents in Korogocho informal settlement in Nairobi, Kenya. Reprod Health. 2020;17(1):79. 32487239 10.1186/s12978-020-00938-3 PMC7268390

[58] Maina BW, Orindi BO, Osindo J, Ziraba AK. Depressive symptoms as predictors of sexual experiences among very young adolescent girls in slum communities in Nairobi, Kenya. Int J Adolesc Youth. 2020;25(1):836–48. 32537261 10.1080/02673843.2020.1756861 PMC7254498

[59] Maina BW, Orindi BO, Sikweyiya Y, Kabiru CW. Gender norms about romantic relationships and sexual experiences among very young male adolescents in Korogocho slum in Kenya. Int J Public Health. 2020;65(4):497–506. 32270236 10.1007/s00038-020-01364-9 PMC7275025

[60] Maina BW, Sikweyiya Y, Ferguson L, Kabiru CW. Conceptualisations of masculinity and sexual development among boys and young men in Korogocho slum in Kenya. Cult Health Sex. 2022;24(2):226–40. 33289439 10.1080/13691058.2020.1829058

[61] Mda P, O’Mahony D, Yogeswaran P, Wright G. Knowledge, attitudes and practices about contraception amongst schoolgirls aged 12–14 years in two schools in King Sabata Dalindyebo Municipality, Eastern cape. Afr J Prm Health Care Fam Med. 2013;5(1):509.

[62] Ninsiima AB, Leye E, Michielsen K, Kemigisha E, Nyakato VN, Coene G. Girls have more challenges; they need to be locked up": a qualitative study of gender norms and the sexuality of young adolescents in Uganda. Int J Environ Res Public Health. 2018;15(2):193. 10.3390/ijerph15020193. 10.3390/ijerph15020193 PMC5858264 29364192

[63] Nyakato VN, Achen C, Chambers D, Kaziga R, Ogunnaya Z, Wright M, et al. Very young adolescent perceptions of growing up in rural southwest Uganda: influences on sexual development and behavior. Afr J Reprod Health. 2021;25(2):50–64. 37585753 10.29063/ajrh2021/v25i2.5

[64] Ortiz-Echevarria L, Greeley M, Bawoke T, Zimmerman L, Robinson C, Schlecht J. Understanding the unique experiences, perspectives and sexual and reproductive health needs of very young adolescents: Somali refugees in Ethiopia. Confl Health. 2017;11(Suppl 1):26. 29163667 10.1186/s13031-017-0129-6 PMC5688399

[65] Selikow TA, Ahmed N, Flisher AJ, Mathews C, Mukoma W. I am not umqwayito’’: a qualitative study of peer pressure and sexual risk behaviour among young adolescents in cape Town, South Africa. Scand J Public Health. 2009;37(Suppl 2):107–12. 19493988 10.1177/1403494809103903

[66] Tchuisseu YBP, Susan K, Ruth K, Dorcus A, Mark R, Christine K, et al. Understanding the sexual and reproductive health rights and experiences of very young adolescents in rural Uganda from the perspectives of emerging adults. Afr J Reprod Health. 2023;27(9):13–21. 10.29063/ajrh2023/v27i9.2 37788306

[67] Zimmerman LA, Koenig LR, Pulerwitz J, Kayembe P, Maddeleno M, Moreau C. The intersection of power and gender: examining the relationship of empowerment and gender-unequal norms among young adolescents in Kinshasa, DRC. J Adolesc Health. 2021;69(1S):S64-71. 34217462 10.1016/j.jadohealth.2021.03.031

[68] Chabata ST, Hensen B, Chiyaka T, Mushati P, Musemburi S, Dirawo J, et al. The impact of the DREAMS partnership on HIV incidence among young women who sell sex in two Zimbabwean cities: results of a non-randomised study. BMJ Glob Health. 2021; 6(4):e003892. 10.1136/bmjgh-2020-003892. 10.1136/bmjgh-2020-003892 PMC8088246 33906844

[69] Sabrina BK, Joseph R, Flavia N, Theresa PW, Fatuma N, Lorraine O, et al. Accuracy of sexual and reproductive health information among adolescent girls: a cross-sectional study. J Pediatr Adolesc Gynecol. 2023;36(3):291–7. 36758720 10.1016/j.jpag.2023.01.218

[70] Potter MH, Font SA. Parenting influences on adolescent sexual risk-taking: differences by child welfare placement status. Child Youth Serv Rev. 2019;96:134–44. 31736530 10.1016/j.childyouth.2018.11.038 PMC6858058

[71] Brown BB. Adolescents’ relationships with peers. In: Lerner RM, Steinberg L, editors. Handbook of adolescent psychology. 2nd ed. Wiley; 2004. p. 363–94. John Wiley & Sons, Inc. 10.1002/9780471726746.ch12.

[72] Steinberg L, Monahan KC. Age differences in resistance to peer influence. Dev Psychol. 2007;43(6):1531–43. 18020830 10.1037/0012-1649.43.6.1531 PMC2779518

[73] Mhlongo S, Mason-Jones AJ, Ford K. Sexual, reproductive and mental health among young men (10–24) in low-and-middle income countries: a scoping review. Front Reprod Health. 2023;5:1119407. 38111839 10.3389/frph.2023.1119407 PMC10725937

[74] WHO. Health for the World’s Adolescents: A second chance in the second decade. Geneva: WHO; 2014.

[75] Gottert A, Pulerwitz J, Weiner R, Okondo C, Werner J, Magni S, et al. Systematic review of reviews on interventions to engage men and boys as clients, partners and agents of change for improved sexual and reproductive health and rights. BMJ Open. 2025;15(1):e083950. 39832970 10.1136/bmjopen-2024-083950 PMC11751930

[76] Onukwugha FI, Smith L, Kaseje D, Wafula C, Kaseje M, Orton B, et al. The effectiveness and characteristics of mHealth interventions to increase adolescent’s use of sexual and reproductive health services in Sub-Saharan africa: A systematic review. PLoS ONE. 2022;17(1):e0261973. 35061757 10.1371/journal.pone.0261973 PMC8782484

[77] Golden SD, Earp JA. Social ecological approaches to individuals and their contexts: twenty years of health education & behavior health promotion interventions. Health Educ Behav. 2012;39(3):364–72. 22267868 10.1177/1090198111418634

