# Supplementary figures and images for: How do social norms influence the sexual and reproductive health-related attitudes and behaviours of very young adolescents in Sub-Saharan Africa? A scoping review
Source: BMC Public Health. 2025 Nov 22;26:7. doi: 10.1186/s12889-025-25736-z (PMC12764140; doi:10.1186/s12889-025-25736-z)

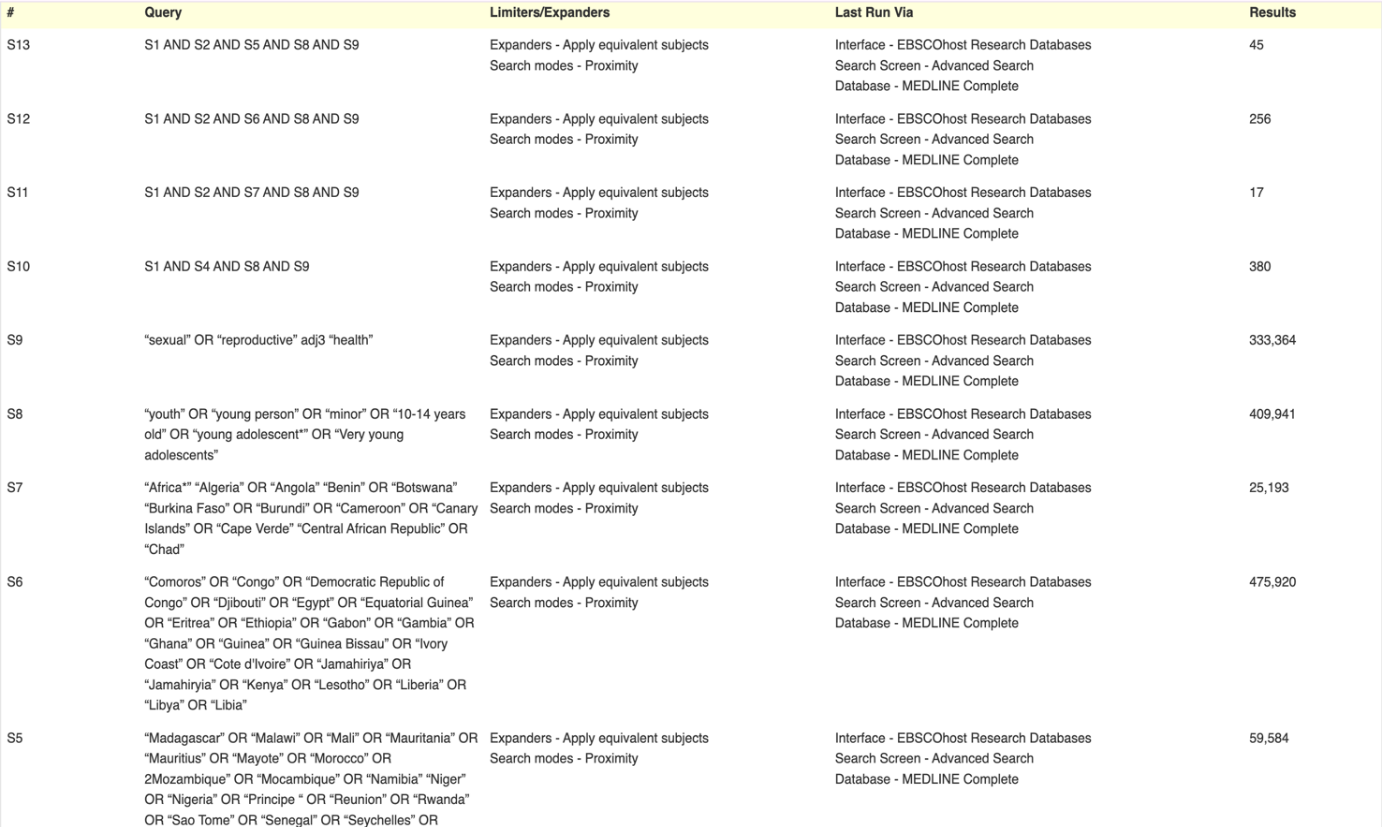


S2 Fig: Medline Search

Supplement: Supplementary file 2 — Supplementary Material 2. [file 12889_2025_25736_MOESM2_ESM.docx]
